# Reproductive health services for populations at high risk of HIV: Performance of a night clinic in Tete province, Mozambique

**DOI:** 10.1186/1472-6963-10-144

**Published:** 2010-05-28

**Authors:** Yves Lafort, Diederike Geelhoed, Luisa Cumba, Carla das Dores Mosse Lázaro, Wim Delva, Stanley Luchters, Marleen Temmerman

**Affiliations:** 1 International Centre for Reproductive Health, University Ghent, Ghent, Belgium; 2 Provincial Health Directorate, Ministry of Health, Tete, Mozambique; 3 South African Centre for Epidemiological Modelling and Analysis, Stellenbosch University, South Africa

## Abstract

**Background:**

Different models exist to provide HIV/STI services for most-at-risk populations (MARP). Along the Tete traffic corridor in Mozambique, linking Malawi and Zimbabwe, a night clinic opening between 4 and 10 PM was established targeting female sex workers (FSW) and long-distance truck drivers (LDD). The clinic offers free individual education and counselling, condoms, STI care, HIV testing, contraceptive services and outreach peer education. To evaluate this clinic model, we assessed relevance, service utilisation, efficiency and sustainability.

**Methods:**

In 2007-2009, mapping and enumeration of FSW and LDD was conducted; 28 key informants were interviewed; 6 focus group discussions (FGD) were held with FSW from Mozambique and Zimbabwe, and LDD from Mozambique and Malawi. Clinic outputs and costs were analysed.

**Results:**

An estimated 4,415 FSW work in the area, or 9% of women aged 15-49, and on average 66 trucks stay overnight near the clinic. Currently on average, 475 clients/month visit the clinic (43% for contraception, 24% for counselling and testing and 23% for STI care). The average clinic running cost is US$ 1408/month, mostly for human resources. All informants endorsed this clinic concept and the need to expand the services. FGD participants reported high satisfaction with the services and mentioned good reception by the health staff, short waiting times, proximity and free services as most important. Participants were in favour of expanding the range of services, the geographical coverage and the opening times.

**Conclusions:**

Size of the target population, satisfaction of clients and endorsement by health policy makers justify maintaining a separate clinic for MARP. Cost-effectiveness may be enhanced by broadening the range of SRHR-HIV/AIDS services, adapting opening times, expanding geographical coverage and targeting additional MARP. Long-term sustainability remains challenging and requires private-public partnerships or continued project-based funding.

## Background

Female sex workers (FSW) are a hard-to-reach, socially and economically vulnerable population with inadequate access to health, social and legal services. They are at increased risk for HIV, sexually transmitted infections (STI) and other sexual and reproductive health and rights problems (SRHR) [1-5]. In Sub-Saharan Africa, sex work is often concentrated along transport corridors, with long-distance truck drivers (LDD) as important clients [6]. Several interventions are targeting FSW and LDD along these corridors, mostly focussing on behaviour change communication, promotion of consistent correct condom use, and improved access to quality STI care and HIV counselling and testing [7-9].

Different models exist to provide targeted HIV/STI services. In large African towns, a model of FSW-specific clinics is often implemented [10-13]. This model is less obvious in settings with smaller concentrations of FSW, such as along transport corridors [14], and projects often opt to improve access to HIV/STI services in existing public or private health facilities [15,16]. Little information is available on the performance of different models and how best to provide these services. Relevance, service utilisation, cost or efficiency are rarely documented.

In 2001, in Northern Mozambique, along the 'Tete corridor' connecting Zimbabwe with Malawi, a 'night clinic' was established specifically targeting FSW and LDD. The clinic is located near a major truck stop, 20 km from the provincial capital Tete, at the edge of Moatize town, and opens at a time considered convenient for the target populations (4-10 PM). Initially the clinic offered STI care and information, education and communication (IEC) on HIV/STI. In a subsequent phase, services were expanded to include contraceptive services and voluntary testing and counselling for HIV and syphilis. A group of 20 peer educators were trained in behaviour change communication and condom distribution. All services are free of charge. The clinic currently operates with health staff and medical supplies from the public sector, with financial and technical support from the International Centre for Reproductive Health (ICRH) in the context of a project funded by the Flemish International Cooperation Agency.

In 2007, concerns were raised about the long-term future of the clinic and it was agreed to quantify the size of the target populations and assess (1) if the model of a separate clinic targeting MARP remained relevant, (2) if the organisation of the services was appropriate to the needs of the target groups, (3) if the services were delivered at a reasonable cost, (4) if there was a demand for additional SRHR services, and (5) if the model was sustainable on the long term.

## Methods

### Mapping and enumeration of FSW and LDD

A mapping and enumeration of sex workers in the two major urban centres near the clinic, Tete and Moatize, was conducted in 2008. First a mapping was done of all establishments and places where FSW recruit clients, and all establishment managers were interviewed using a structured questionnaire. Topics covered included characteristics of FSW and clients at the establishment, time of peak sex work activity and characteristics of sex work. Based on this information, the day of the month and the hours of the day of peak FSW activity were identified and during that period the number of FSW was enumerated. The two towns were divided in 11 areas, where, simultaneously, pairs of peer educators counted FSW actively recruiting clients, identified through predefined criteria of behaviour and appearance. Data were analysed using MS-Excel. In addition, an enumeration was conducted in 2007-2008 of the number of trucks that stayed overnight at the nearby truck stop.

### Key informant interviews

Eight key informants, from the Provincial and District health authorities, the Provincial AIDS Council, non-governmental organisations and a mining company, were individually face-to-face interviewed using semi-structured questionnaires. The 3 clinic staff and 17 peer educators were interviewed in two separate groups. Topics covered included the type of relationship with the clinic, opinions about the performance of the clinic (strengths, weaknesses, opportunities and threats), suggestions for improvement, opportunities for collaboration and suggestions for ensuring long-term sustainability. Notes were taken by the interviewer and transcribed the same day.

### Focus group discussions

To obtain client perspectives, six focus group discussions (FGDs) were held at the beginning of 2008 using a discussion guide, one with Mozambican FSW (n = 8), one with Zimbabwean FSW (n = 8), two with Mozambican LDD (n = 8 and 10) and two with Malawian LDD (n = 7 and 7). The project peer-educators recruited known FSW from bars and hotels near the night clinic. LDD were recruited from the nearby truck stop and through two major transport companies. Discussions were held at the clinic or in a private room at a restaurant. All participants gave verbal informed consent. Topics discussed included experiences with the clinic, a comparison of the clinic to other health services and suggestions to improve the services. The discussions were guided by a trained moderator in the language chosen by the participants (Nyungue, Chichewa or Shona) and lasted approximately one hour. Notes were taken in Portuguese, which the moderator and note-taker together transcribed directly afterwards.

### Analysis of clinic statistics and costs

Routinely collected information of the clinic between September 2004 and June 2009, including the number of clients, socio-demographic, behavioural and clinical characteristics, motivation for the visit, and operational project costs, was entered in MS-Excel and monthly averages were calculated.

### Ethical clearance

The study was conducted according to international ethical standards. The Mozambican ethical guidelines only require ethical clearance for studies that collect individual data from human subjects. All of the applied methods, including the focus group discussions, are not considered as such and no ethical clearance was needed.

## Results

The results of the different data collection methods were triangulated to assess (1) relevance of the model, (2) service utilisation, (3) service delivery efficiency and (4) long-term sustainability.

### Relevance of the model

#### Size of the target populations

During the mapping, a total of 221 sites where FSW recruit clients were identified in the two towns, 218 establishments (bars or hotels) and 3 outdoor areas (streets). The periods of peak FSW activity were said to be the end and the beginning of the month, on Friday and Saturday nights, between 6 PM and midnight. The most important clients of FSW were said to be merchants, students, truck drivers and public servants.

An enumeration of FSW between 7:30 and 9 PM on a Friday night at the beginning of the month in all establishments counted a total of 4,415 FSW active during that period, 1,158 in Moatize and 3,257 in Tete town. The estimated number of women aged 15-49 years in the two towns is 48,500 and the estimated ratio FSW/women aged 15-49 years is therefore 9.1%.

Between 13 December 2007 and 4 January 2009, 19,210 trucks were counted staying overnight at the truck stop near the clinic, giving an average of 66 trucks per counted night. About half (54%) was of Malawian origin, 20% of Zimbabwean, 16% of Mozambican and 10% of other origin.

#### Coherence with national policies

All interviewed informants endorsed the concept of a separate SRHR clinic for MARP and the need to expand these services to other parts of the province. However, they all agreed that currently this type of services cannot be offered by the public health system, because the national health policies do not recognise separate health facilities for MARP.

#### Client satisfaction

Both FSW and LDD participating in the FGD, of all nationalities, reported satisfaction with the night clinic and mentioned the good reception by the health staff, the prompt attendance, the proximity and the services free of charge as most important strengths of the clinic in comparison to other, formal and non-formal, health care providers.

Malawian LDD: *'In relation to the clinic's reputation, I think it is good. You know why? Because it is free and because of the staff that attends us. Each time I come here, they are always ready. I pass here often.'*

Zimbabwean FSW: *'... Nothing compares to this clinic. The information given is clear and simple while in the hospitals where I went it takes a long time, you spend money and sometimes you are rapidly dispatched because of the crowdedness and not adequately treated...'*

Mozambican LDD: *'... the clinic is very well situated, it is near our depot, is easy to reach, and in little time, I like the attendance, and the services that are offered are excellent...'*

Stigmatisation at the regular health services was in particular reported by Zimbabwean FSW.

Zimbabwean FSW: *'...In other hospitals we are badly attended because we are Zimbabweans. They say they don't understand our language and that we are prostituting men who are their husbands. We have to undergo all sort of humiliation in addition to spending money...'*

Negative feedback on the clinic's activities was given by a Zimbabwean FSW who reported a stigmatising attitude by the peer educators. An LDD participant suggested using male peer educators for LDD, because female peer educators might be perceived as FSW.

Zimbabwean FSW: *'...Who I really don't like are the peer educators. They sometime mock us and say that the Zimbabweans came to steal our bread. Here it is the rule of survival. We are a lot. Sometimes there is not a lot of business and we argue over the same client...'*

Malawian LDD: *'I propose to have male activities as well, for the counselling of truck drivers. Because these female activists, many of us we end up seducing them and so they make another profit.'*

Some FSW participants complained of security problems and violence against them.

Zimbabwean FSW: *'Here in Moatize we are facing violence against sex workers. We need peer educators patrolling in the parking station of the truck drivers'*

### Service utilisation

#### Clinic attendance and profile of attendees

The average number of monthly clinic visits gradually increased from 206 in 2005 to 475 in 2009 (Table 1). Monthly STI consultations increased from 43 in 2004 to 109 in 2009, the large majority (91.8%) being first visits. Consultations for other reasons were disaggregated by IEC, including HIV and syphilis testing, and others until 2007 and by HIV/syphilis counselling and testing, contraception and others afterwards. HIV testing was introduced in 2006 and the average number of tests performed each month increased to 115 in 2009. Consultations for contraceptive services were initially rare, but steeply increased in the last two years to 205 per month. The number of LDD monthly contacted by the peer educators increased from 367 in 2004 to more than thousand in 2005 (1580) and 2006 (1180). The number of distributed condoms increased from 3151 in 2004 to 9200 in 2009. During 2005 and 2006 the peer educators collected information on the country of origin of 31,569 LDD. About half (46%) was from Malawi, 24% from the Mozambican port Beira and 23% from Zimbabwe.

**Table 1:** Average number of monthly client visits and peer educator activities, 2004-2009.

Average nr of monthly visits	**2004**^**1**^	2005	2006	**2007**^**2**^	2008	**2009**^**3**^
Total clinic visits	NA	206	212	257	309	475

STI client visits	43	66	67	115	89	109
Women	24	34	35	74	62	75
Men	19	33	31	41	27	35
FSW	NA	24	30	67	NA	NA
LDD	NA	20	10	28	NA	NA
Individual education and counselling	NA	112	119	121	73	115
Women	NA	69	65	103	NA	NA
Men	NA	43	53	18	NA	NA
FSW	NA	28	33	9	NA	NA
LDD	NA	41	28	65	NA	NA
Tested for HIV^4^	NA	NA	54	54	73	115
% positive	NA	NA	28.7%	26.9%	17.5%	26.6%
Tested for syphilis^4^	NA	NA	86	75	53	37
Other client visits	NA	28	26	21	147	251
Contraception client visits	NA	NA	NA	NA	120	205

Peer educator activities						
LDD contacted	367	1,580	1,180	NA	NA	NA
Condoms distributed	3,151	2,801	8,390	NA	8,289	9,200

^1 ^Data were only available for the period September-December. ^2 ^Data on STI clients and HIV/syphilis testing were available until October. Data on individual counselling were available until April. Data of the months of June to August were not included because the clinic was mostly closed during that period. ^3 ^Data were only available for the period January-June. ^4 ^HIV and syphilis testing initiated in March 2006. All tested clients are counted as IEC clients as well.

During 2005-2006 several socio-demographic and behavioural characteristics were collected from clinic clients (Table 2). Half of STI clients (49%) and slightly more than half of individually counselled clients (58%) were female and the large majority of counselled clients (80%) and almost all STI clients (97%) were 20 years or older. In both groups slightly more than half (59% of STI clients and 57% of counselled clients) were Mozambicans. FSW and LDD represented 61% of STI clients and 57% of counselled clients; 86% of STI clients and 93% of counselled clients reported more than one partner in the past 12 months and respectively 68% and 76% reported the use of a condom at last sexual contact.

**Table 2:** Socio-demographic and behavioural characteristics of clinic attendees, 2005-2006.

Characteristic	STI clients (N = 1,768)		Individual education and counselling (N = 2,760)	
	n	%	n	%
Sex				
Male	867	49.0%	1,606	58.2%
Female	901	51.0%	1,154	41.8%
Age				
<15 years	0	0.0%	1	0.0%
15-19 years	51	2.9%	541	19.6%
>= 20 years	1,717	97.1%	2,219	80.4%
Country of origin				
Mozambique	1,062	58.5%	1,566	56.7%
Zimbabwe	609	33.5%	886	32.1%
Malawi	129	7.1%	270	9.8%
Other	16	0.9%	38	1.4%
Occupation				
FSW	650	39.0%	731	26.6%
LDD	360	21.6%	831	30.3%
Higher education students	153	9.2%	247	9.0%
School students	118	7.1%	267	9.7%
Teachers	61	3.7%	126	4.6%
Others	325	19.5%	543	19.8%
Education				
None	336	24.3%	107	3.9%
Primary	687	49.8%	913	33.1%
Secondary	357	25.9%	1,740	63.0%
Nr of partners in last 12 months				
<1	178	13.7%	225	8.3%
1 to 2	312	24.1%	869	32.1%
3 to 5	306	23.6%	411	15.2%
>6	501	38.6%	1,203	44.4%
Used condom at last sex act				
Yes	716	68.0%	2,108	76.4%
No	337	32.0%	652	23.6%

### Efficiency

#### Costs

The capital and operational costs of the clinic are partly borne by the Mozambican government. These comprise the plot where the clinic is situated, the regular salaries and benefits of the clinic's three nurses and the drugs and medical supplies. All other costs are financed by the project, including the infrastructure (two containers), medical equipment and furniture, utilities (water and electricity), security (three guards), over-time compensation for the nurses, training costs, educational materials and some condoms, and all peer educator costs. The project operational costs for the period 2004-2009 are summarised in Table 3. The monthly cost borne by the project fluctuated initially around 950 US$/month, the human resource cost (overtime fees, guard salaries and peer educator incentives) being responsible for the largest proportion (87% of the total costs in 2007). After 2007 the cost increased to approximately 1400 US$ per month due to an adjustment of the watchmen's salaries and peer educators incentives.

**Table 3:** Monthly clinic costs borne by the project, 2004-2009.

Average monthly cost (US$)	9/2004- 7/2005	8/2005- 7/2006	8/2006- 6/2007	9/2007- 12/2007	1/2008- 12/2008	1/2009- 6/2009
Overtime payment health staff	664	711	374	383	1358	1408
					
Security (watchers' salary)			217	298		
						
Peer educators' incentives				295		
		
Consumables	230	165	(Not available)	40		
						
Maintenance				10		

Utilities	61	66	79	92		

Total	956	942	670	1119	1358	1408

#### Opening hours

The most important factor to increase the accessibility to the clinic was said to be a revision of the opening time of the clinic. Most focus groups agreed that the hours between 4-10 PM were not the most convenient and expressed a desire to open the clinic also during day time and during the weekend.

Mozambican FSW: *'...The opening times should be from 12 to 18 h, because from 18 h onwards we start meeting our clients...'*

Malawian LDD: *'...I think the opening times should be changed to allow that we can be attended whenever we are passing through. Also during weekends....' '...I agree with my colleague, but I propose that the clinic is open from 10 h to 22 h...'*

Mozambican FSW: *'...Change [the opening times] because there are bandits here. From 18 h onwards we don't feel safe because of these bandits...others think wrongly that we come this way looking for men. Others think we have cell phones and money they can steal from us. One day we'll die because of cell phones. A better time is 14 h and closing at 18 h, or start at 7h30 and stop for example at 12 h, another starts and stops at 17 h'*.

#### Scope of services

FGD participants were generally satisfied with the range of offered services. Some suggested offering treatment for common, not SRHR-related, illnesses, but others did not agree fearing overcrowding.

Malawian LDD: *'... I also would like that other illnesses would be treated at the clinic. For example malaria, because a lot of us get malaria in Beira where we sleep in the open and in the trucks and we are exposed to mosquitoes... That is important, yes. Malaria should be among the list of illnesses that are treated here.'*

Zimbabwean FSW: *'I don't want to contradict that what my friend said [about treating malaria and diarrhoea], but I think it is better as it is, a treatment specifically for STI and HIV/AIDS tests. Because if the attendance would be expanded to other illnesses, we wouldn't have this luxury, and everyone would come to the clinic because it is free of charge, and there would be shortage of medicines, and the waiting time would be long, and we are the ones that would suffer.'*

Some suggested providing more educational tools, such as films, others suggested offering ARV treatment to those who tested HIV positive.

Mozambican FSW: *'We would also like that antiretrovirals would be distributed here.... [Now] It is necessary to go there [at the hospital], others already don't have the strength to go there, they are already very sick... When they do the test here and it is positive, they say to go to the hospital. Here they don't give antiretrovirals.'*

### Long-term sustainability

Key informants reported that the public health system is currently contributing as much as possible within the national health policies. It provides the staff and all medical supplies, but it cannot finance the clinic and the peer education activities. A continued support by a non-governmental agency was said to be necessary. Funding can only be guaranteed through project-based funding or through private-public partnerships. The only two valid alternatives for long-term funding at the time of the assessment were (1) continued support through an externally funded project, or (2) development of a public-private partnership between the major mining companies and the Mozambican Government.

## Discussion

The main question posed in the evaluation was if a separate clinic for the provision of HIV and other SRHR services for FSW and LDD is justified in the context of the Tete corridor. Many projects in similar contexts have opted to enhance access to existing health services instead of creating new services [8,15,16]. The main arguments against separate services are that they are more expensive and potentially less sustainable. They appear justified in areas with a very high concentration of FSW, such as in large cities, but the model is more contested in settings such as along transport routes, where the numbers are believed to be smaller. Our assessment is a case study of an existing freestanding clinic and we do not know how reducing barriers at existing public health clinics would have performed. Nevertheless, our results provide some arguments to justify maintaining a separate facility in the context of the Tete corridor.

Firstly, the size of the target population was much larger than anticipated and comparable to what is found in large urban areas. The ratio of FSW/women of reproductive age is higher than what is found in most settings in Sub-Saharan Africa [17].

Secondly, all FGD participants strongly expressed their satisfaction with the clinic and preferred it above other health services. The arguments mentioned were that the clinic is much easier accessible, both because of its location and because of the limited waiting times, the reception is much better, confidentiality is guaranteed and the services are free. This coincides with the results of a cross-sectional survey conducted among FSW and LDD along the corridor in 2006 in which accessibility, confidentiality and free treatment were also the three most common reasons mentioned to prefer the night clinic above other health facilities [18]. It is also in accordance with the findings of a study along the Tanzania-Zambia highway assessing four different models to deliver STI care to women engaging in transactional sex [14]. In two of the models STI care was strengthened at existing health services and in two STI care was offered outside health facilities or at times other than the normal clinic hours. Clinic attendance was the highest in the latter models. A similar experience was documented in Kinshasa where attendance of FSW at a specialized clinic dropped dramatically when the clinic was opened to the general population [19].

A surprising finding was that the opening times, which were assumed to be appropriate for the target populations, did not appear to be so. Most FSW participating in the FGD expressed a desire to open the clinic earlier, and the later evening hours (after 8 PM) were said to be inappropriate for security reasons and because at that time they are already at work. Most LDD preferred opening hours during the day and weekends so that they can stop while passing the clinic.

Thirdly, although the national health system never adopted the concept of clinics specifically targeting MARP, all interviewed health officials completely endorsed the model end expressed a desire to strengthen and expand it. This is further shown by the willingness to contribute as much as possible within the health system regulations, such as by providing the necessary drugs and medical supplies. The need of specific services for MARP is also increasingly recognised at central level in Mozambique [20] and it might only be a matter of time before a policy is developed.

The frequentation of the clinic was initially rather low, but increased substantially the last two years mostly because of the increase in contraceptive services use. At the end of the assessment the number of daily visits to the clinic was about 21. To what extent the target populations are currently making use of the clinic is difficult to assess. Most FGD participants attended the clinic, but they were recruited from the immediate neighbourhood. The 2006 survey asked for treatment seeking behaviour for the last STI symptoms, but had not listed the night clinic among the options. Even so, 16% of FSW and 7% of LDD reported the night clinic under 'others' [18]. The number of consultations appears still low in comparison to the size of the target population and it is probable that FSW operating further away from the clinic do still not know about its existence.

The cost of the clinic is relatively high. No figures were available on the start-up capital cost (two containers, medical equipment and training) and only the operational cost was analysed. Some of the operational cost, mainly the salaries of the health staff and the medical supplies, is borne by the national health system. This cost is not specific to the model of a stand alone clinic and is probably comparable to the cost of services for MARP integrated in existing health facilities. The additional operational cost caused by the stand alone model is borne by the project and comprises the cost of staff overtime, security, utilities and peer education. The total project cost varied from slightly under 1000 US$ per month at the beginning to around 1400 US$ per month currently, or about 4.5 US$ per client visit. This is high when taking into account Mozambique's limited resources for health (a per capita total expenditure on health of 17 US$ in 2006). The long-term sustainability of the clinic remains therefore a challenge. The total cost can currently not be borne by the national health system, and external funding and support in the management of the services are necessary. A possible way out are long-term private-public partnerships with the mining companies that are currently initiating the exploitation of the coal mines in Moatize. Many of the companies' staff are at high risk for HIV and other STIs and therefore represent a potential target population for the clinic.

The effect of the model in terms of number of HIV infections averted, unwanted pregnancies averted and DALY's gained is not known and it is not possible to appreciate the cost-effectiveness. However, HIV prevention among FSW is generally highly cost-effective [21-23] because of the role FSW play as core transmitters. A relatively large incremental cost of stand alone clinics effectively reaching FSW and their clients may therefore be justified. A further analysis of the cost-effectiveness of the model, in comparison to integrated services, is indicated and more transparency is needed in expenditures on SRHR services for MARP in different settings to allow cross-model comparison.

The cost per visit can be reduced by enlarging the scale and expanding the services to other FSW and MARP at a greater distance and by better targeting FSW clients, including populations that appear to be important clients such as merchants and students. Attendance can be further increased by broadening the range of services offered. Although the clinic has expanded its services from purely STI care, IEC and condom distribution, to include testing and counselling and contraceptive services, it is still far from being a comprehensive SRHR clinic. When FGD participants were asked if they had any suggestions for extra services to be offered at the clinic, the highest need appeared to be access to treatment for general illnesses. Other participants were fully aware, however, that providing these services at the clinic would transform it into a general health clinic. Few participants requested extra SRHR services, such as for harm reduction or empowerment. This is probably because of the open way the question was asked and because FSW might not be aware of what services are possible. Indirectly, the need for extra services became clear, such as by the mentioning of the frequent occurrence of violence. A lack to access to antiretroviral treatment was also mentioned and there is a need to strengthen the prevention-care continuum.

A substantial increase in the number of clients has the risk of compromising some of the factors that lead to client satisfaction, such as confidentiality or limited waiting times. The current average number of clients per hour is about 3.6 and there is little potential for further increase without expanding the opening hours or establishing outreach sites. A careful monitoring of client satisfaction is indicated.

## Conclusion

The current model for improving access to quality SRHR services for FSW and other MARP along the Tete corridor, Mozambique, is challenged by high incremental operational costs but remains a valid model overall. The performance can be enhanced by broadening the range of SRHR-HIV/AIDS services, adapting the opening times, expanding the geographical coverage, targeting additional MARP and further improve the quality of the services. Long-term sustainability can currently only be ensured by private-public partnerships or continued project-based funding.

## Competing interests

The authors declare that they have no competing interests.

## Authors' contributions

YL was the lead investigator during the performance assessment, developed the overall design of the assessment, the triangulation analysis and drafted the manuscript. He also developed and carried out the key informant interviews and the analysis of the clinic statistics and costs. DG greatly contributed to the overall design of the assessment and developed and carried out the focus group discussions and the mapping and enumeration. She helped to draft the manuscript. LC and CDML substantially contributed to the design and the preparation of the assessment and its different components. SL contributed to the design of the assessment, assisted in the triangulation analysis and helped to draft the manuscript. WD assisted with the analysis of the clinic statistics and costs and the revision of the manuscript. MT substantially contributed to draft the manuscript. All authors read and approved the final manuscript.

## Pre-publication history

The pre-publication history for this paper can be accessed here:

http://www.biomedcentral.com/1472-6963/10/144/prepub
